# Implantable Cardioverter-Defibrillator (ICD) Lead-Induced Septal and Left Ventricular Perforation in Hypertrophic Cardiomyopathy: A Case Report

**DOI:** 10.7759/cureus.103333

**Published:** 2026-02-10

**Authors:** Philippos Alexiou, Christos E Ballas, Christos Alexiou

**Affiliations:** 1 Medical School, Aristotle University of Thessaloniki, Thessaloniki, GRC; 2 Department of Cardiac Surgery, University Hospital of Ioannina, Ioannina, GRC

**Keywords:** cardiac tamponade, hypertrophic cardiomyopathy, implantable cardioverter-defibrillator, lead perforation, left ventricular perforation, surgical lead extraction

## Abstract

Left ventricular (LV) perforation caused by an implantable cardioverter-defibrillator (ICD) lead is a rare but potentially fatal complication. We describe the case of a 34-year-old man with hypertrophic cardiomyopathy who underwent single-chamber ICD implantation for primary prevention of sudden cardiac death using an active-fixation transvenous lead positioned in the right ventricular septum. Four days after implantation, he developed acute chest pain, dyspnoea, hypotension, with clinical signs of evolving cardiac tamponade. Transthoracic echocardiography demonstrated a moderate circumferential pericardial effusion, while device interrogation revealed abnormal pacing parameters, prompting emergency pericardiocentesis with immediate haemodynamic stabilization. Computed tomography was subsequently performed to accurately delineate lead position and confirmed perforation through the interventricular septum with extension into the LV free wall, a finding that could not be fully characterized by echocardiography alone. The patient was transferred to a tertiary centre with continuous cardiac surgical support, where lead extraction and surgical repair of the myocardial defects were successfully performed using pledgeted sutures. The postoperative course was uneventful and follow-up imaging confirmed complete resolution of the pericardial effusion. Unlike the typical pattern of right ventricular free-wall perforation, this case involved transseptal migration of an ICD lead with left ventricular involvement, highlighting that hypertrophic myocardium does not preclude this life-threatening complication and underscoring the importance of early recognition and individualized management.

## Introduction

Permanent cardiac device implantation is a widely performed procedure for the management of arrhythmias, conduction disorders and heart failure (advanced device-based intervention) [1,2]. It is generally a safe procedure. One of the most dangerous lead-related complications is cardiac perforation, which can be fatal. However, with an estimated incidence of just 0.1% to 0.8%, it remains relatively rare [3]. The classification of perforation is dependent on the time of symptom onset and thus can be categorized as acute (presenting within 24 hours), subacute (within 30 days), or chronic (more than 30 days after implantation) [4].

From a pathophysiological perspective, lead perforation occurs as a result of mechanical myocardial penetration by the lead tip, which may be facilitated by excessive lead tension, active fixation mechanisms, progressive myocardial erosion, or repetitive cardiac motion over time. Most cases involve the right ventricle (RV) [5]. The perforation of the left ventricular (LV) apex is exceedingly rare, while septal perforation with extension into the LV has been reported only in isolated cases.

Linked factors that increase the chances of myocardial perforation can be broadly divided into patient- and device-related factors. Patient-related factors include female gender, older age, thin ventricular wall, corticosteroid use, chronic obstructive pulmonary disease (COPD), low body mass index (BMI), and congestive heart failure. Device- and procedure-related factors include the use of active fixation leads, over-torquing during lead deployment, and lead tip placement at the RV apex or apical septum [2,6-9].

This case describes a septal and LV perforation in a 34-year-old man with a history of hypertrophic cardiomyopathy, following implantation of a cardioverter-defibrillator lead for primary prevention of sudden arrhythmic cardiac death. Although hypertrophic myocardium has traditionally been considered protective due to increased wall thickness, focal myocardial vulnerability, altered fiber architecture, and increased stiffness in hypertrophic cardiomyopathy may predispose to perforation, indicating that hypertrophic myocardium is not absolutely protective [3]. Hence, we discuss the management of this complication and provide a literature review of similar cases.

## Case presentation

A 34-year-old man with a history of hypertrophic cardiomyopathy was referred to the cardiac surgery department of a tertiary hospital in Western Greece for urgent evaluation. The patient recently underwent cardioverter-defibrillator (ICD) implantation for primary prevention of sudden arrhythmic cardiac death. His past medical history was otherwise unremarkable. He had no history of chronic pulmonary disease, ischemic heart disease, connective tissue disease, or prior cardiac surgery. His BMI was normal (23.4 kg/m²) and his medication history showed he was not receiving corticosteroids or anticoagulants. Pre-implant transthoracic echocardiography demonstrated increased interventricular septal thickness (15.5 mm). No antithrombotic therapy was administered during the peri-implantation period.

The ICD implantation was performed four days prior to admission to our department, while hospitalized at the department of cardiology of another tertiary hospital, without 24/7 cardiac surgery coverage. A single-chamber ICD with an active-fixation RV lead was implanted via the left subclavian vein. The lead was positioned in the RV septum. The patient’s intraoperative course was normal without complications and the initial postprocedural device parameters were within normal ranges.

Two days after implantation, while the patient was still hospitalized in the hospital where the ICD implantation took place (two days before his transfer to our hospital), he developed acute chest pain, new-onset dyspnoea, and marked generalized weakness. Over the few next hours, the patient’s symptoms worsened, with mildly decreased level of consciousness, whereas physical examination revealed hypotension (blood pressure 90/55 mmHg), tachycardia (heart rate 110 beats per minute), and tachypnea (27 respirations per minute). Furthermore, it was observed that the patient had jugular venous distension, muffled heart sounds, and cool extremities, signs suggestive of evolving cardiac tamponade. Oxygen saturation was 95% (FiO_2_=21%).

Electrocardiogram revealed sinus tachycardia without signs consistent with an acute ischemic event. Laboratory tests revealed mild leukocytosis and elevated inflammatory markers, while cardiac troponin levels were minimally elevated, without significant change compared to the preprocedural value. The key laboratory findings at the time of cardiac tamponade diagnosis are summarized in Table 1. Device interrogation demonstrated abnormal pacing parameters with increased pacing thresholds and reduced sensing amplitude. The transthoracic echocardiogram revealed a moderate circumferential pericardial effusion with early diastolic right atrial collapse, findings consistent with tamponade.

**Table 1:** Key laboratory findings at the time of cardiac tamponade diagnosis (POD 2) compared with pre-procedural values PreOP: Preoperatively, POD: Postoperative day, HCT: Hematocrit, HGB: Hemoglobin, PLT: Platelet count, Creat: Creatinine, AST: Aspartate transaminase, ALT: Alanine transaminase, HsTN-I: High-sensitivity troponin-I, N/A: Not available

Laboratory Test	PreOP	POD 2	Reference Range	Units
HCT	45.3	39.2	34-47	%
HGB	14.6	12.7	12-15.5	g/dl
PLT	192	148	140-450	10^3^/μl
D-Dimers	N/A	2.21	0-0.5	μg/ml
Urea	39	56	11-54	mg/dl
Creat	0.68	1.18	0.6-1.2	mg/dl
AST	32	40	10-35	IU/l
ALT	30	32	10-35	IU/l
HsTN-I	9.2	282	0-11.6	pg/ml

**Figure 1:**
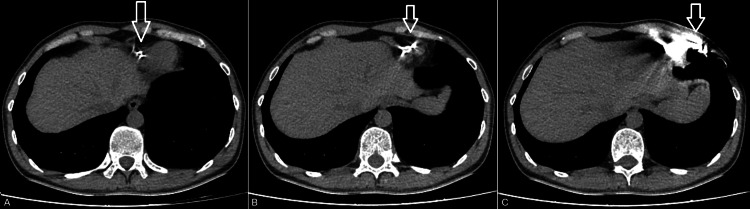
Computed tomography of the chest demonstrating ICD lead perforation through the interventricular septum (arrow, A), extension into the left ventricular free wall (arrow, B), and final exit into the pericardial cavity (arrow, C). ICD: Implantable cardioverter-defibrillator

**Figure 2:**
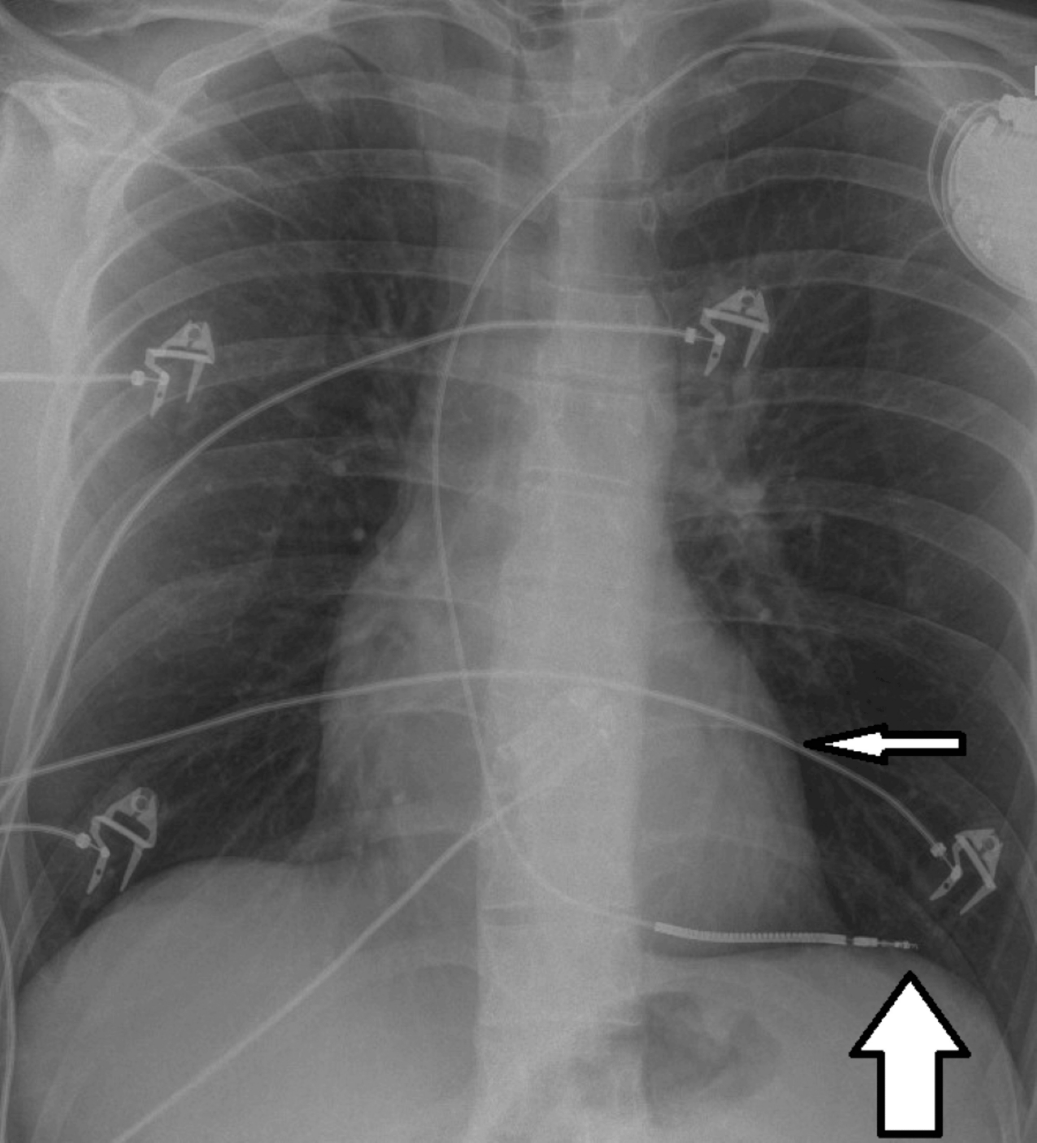
Chest radiograph demonstrating an ICD lead outside the cardiac silhouette (large arrow), with the cardiac silhouette indicated by a small arrow. ICD: Implantable cardioverter-defibrillator

Following this finding, it was decided to perform an emergency pericardiocentesis under echocardiographic guidance, from which 400 ml of haemorrhagic pericardial fluid was successfully drained, resulting in immediate hemodynamic improvement, with stabilization of blood pressure and relief of patient’s symptoms. A pericardial drain was left in situ for continuous monitoring. The procedure followed by a contrast-enhanced computed tomography of the chest in which perforation of the ICD lead through the interventricular septum with extension into the LV free wall was found (Figure 1). Chest radiography also revealed the ICD lead positioned outside the cardiac silhouette (Figure 2).

Despite initial stabilization, the persistence of lead malposition and the high risk of ongoing myocardial injury necessitated definitive surgical management. Surgical extraction was favored over transvenous lead removal due to the confirmed transseptal and LV perforation, the recent onset of cardiac tamponade, and the need for direct myocardial repair.

**Figure 3:**
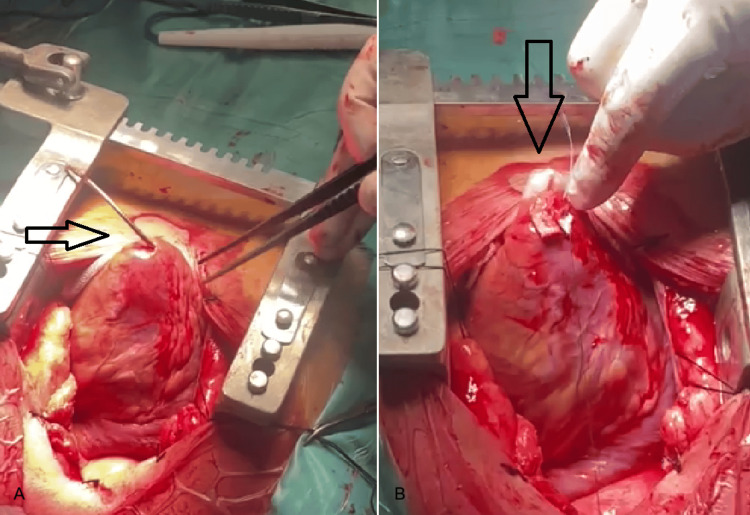
A. Perforation of the RV septum and LV wall during ICD lead implantation (arrow). Intraoperative photograph from the subsequent open-heart surgery. Β. Intraoperative photograph showing myocardial defect repair with pledgeted sutures (arrow). RV: Right ventricular, LV: Left ventricular, ICD: Implantable cardioverter-defibrillator.

The patient was transferred to our hospital, where he underwent open surgical lead extraction under general anaesthesia. Intraoperatively, the ICD lead was found to traverse the RV septum and perforate the LV wall (Figure 3A). The distal tip of the lead was transected and removed. The remaining portion of the lead was left freely within the RV, with the intention of elective removal at a second stage, concomitant with reimplantation of a new ICD lead. This staged approach was selected to minimize operative time and myocardial manipulation in the acute setting following tamponade. The myocardial defects were repaired using pledgeted sutures (Figure 3B). The postoperative course was uneventful. Follow-up echocardiography demonstrated complete resolution of the residual pericardial effusion. The patient was discharged, in stable condition, on postoperative day seven.

At three-month follow-up, he remained asymptomatic with no recurrence of pericardial effusion. Also, within this period, a new ICD lead was implanted without perioperative complications.

Written informed consent was obtained from the patient for both the procedure and the publication of this case.

## Discussion

Ventricular perforation by an ICD lead is a life-threatening complication. It is most commonly seen in the RV, whereas cases that involve the migration of the lead through the interventricular septum and into the LV are exceedingly rare. It can trigger very serious events, such as pericardial effusion, cardiac tamponade, and haemothorax [2,6,8,9]. It often has a subtle presentation. Patients may appear asymptomatic or present with symptoms ranging from chest pain to syncope [2,5,6,10-12]. This underlines the need for clinicians to heighten their awareness, even in cases that initially appear straightforward [4,13].

After a comprehensive review of 12 known cases of lead perforation through the LV, summarized in Table 2, several observations can be made. Female sex has traditionally been considered a risk factor for lead perforation, possibly due to smaller cardiac dimensions and thinner myocardial tissue, particularly in elderly patients [8]. In our analysis, however, the sex distribution is nearly even. With the inclusion of our patient, the reported cases comprise seven men and six women, suggesting that sex alone may not be a dominant risk factor when compared with age, myocardial characteristics, and implantation technique or applied force [2,6-8].

**Table 2:** The 12 previously reported cases of left ventricular perforation caused by an implantable cardiac device lead, the patients’ clinical presentation, and the management strategies applied. ICD: Implantable cardioverter-defibrillator; RV: Right ventricular, LV: Left ventricular, M: Male, F: Female, PM: Pacemaker, N/A: Not available.

References	Sex	Age (years)	Lead type	Lead model	Lead position	Time from implantation to lead complication	Symptoms	Lead migration	Management	New lead position
Bao et al. [4]	M	61	Active fixation PM lead	N/A	N/A	3 days	Persistent chest pain	Septum, LV wall, pericardium	Surgical extraction	LV myocardium
Satomi et al. [5]	M	84	Active fixation PM lead	Select secure 3830-69, Medtronic	RV septum	2 days	Syncope	Septum, LV wall	Surgical extraction	RV septum
Narain et al. [9]	F	74	Active fixation PM lead	N/A	N/A	3 days	Left sharp chest pain,	Septum, LV wall, pericardium	Surgical extraction	RV apex
Iribarne et al. [10]	F	69	Active fixation lead	CapSureFix 5076-52, Medtronic	RV septum	2 weeks	None	Septum, LV wall	Surgical extraction	Epicardial
Viscogliesi et al. [11]	F	84	Active fixation PM lead	N/A	N/A	<1 day	Chest pain	Septum, LV wall	Surgical extraction	N/A
Mililis et al. [12]	M	73	Active fixation PM lead	Beflex lead-microport	RV septum	6 days	syncope	Septum, LV wall	Surgical extraction	RV septum
Hernandez et al. [13]	F	84	Active fixation PM lead	CapSureFix Novus Mri SureScan 5076-52, Medtronic	RV septum	8 days	Chest pain, syncope, bradycardia	Septum, LV wall	Surgical extraction	RV septum
Marazzato et al. [14]	F	78	Active fixation PM lead	Solia S 60, Biotronik	RV septum	2 months	Chest pain, syncope	Septum, LV wall	Surgical extravtion	Epicardial
Nishinarita et al. [15]	M	70	Active fixation ICD lead	SelectSecure, Medtronic	RV septum	2 months	Shortness of breath (for 2weeks)	Septum, LV wall	Surgical extraction	RV apex
Migliore et al. [16]	M	52	Active fixation ICD lead	Linox SD 65/16, Biotronik	RV apex	12 days	Asymptomatic	Septum, LV wall	Surgical extravtion	Epicardial
Huang et al. [17]	M	92	Active fixation PM lead	Surescan 5076-58, Medtronic	RV apex	2 days	Chest pain, syncope	Septum, LV wall	Transvenous extraction	RV septum
Higashimoto et al. [18]	F	86	Passive fixation PM lead	CapSure Sense Model 4574 and 4074, Medtronic	N/A	9 years	Body temperature rise, general fatique, bradycardia	Septum, LV wall, pericardium	Surgical extraction	Epicardial

Age appears to play a significant role in LV free-wall perforation, with most reported cases involving elderly patients (mean age 75.5 years). Age-related myocardial thinning and degenerative changes may increase susceptibility to mechanical injury [10]. Although our patient was a 34-year-old man and represents one of the youngest patients reported to date, this case demonstrates that younger patients are not immune to lead perforation. Therefore, all patients should be assessed with the same degree of vigilance, without excluding lead perforation solely based on young age.

It is also important to distinguish between pacemaker and ICD leads, as ICD leads are generally thicker, stiffer, and require higher fixation forces compared with standard pacing leads. These mechanical properties may increase the risk of myocardial injury, particularly when excessive force or over-torquing is applied during active-fixation lead deployment [5,8].

In the reviewed cases, a substantial proportion of leads were positioned in the RV septum rather than the RV apex [5,10,12-17]. Septal pacing is often favored due to its more physiological ventricular activation and the greater myocardial thickness of the interventricular septum compared with the RV free wall [10]. In hypertrophic cardiomyopathy (HCM), septal thickness is typically increased relative to non-hypertrophic hearts, which may create a false sense of procedural safety. However, despite increased wall thickness, myocardial fiber disarray, microvascular ischemia, and regional heterogeneity characteristic of HCM may result in focal areas of vulnerability, rendering the myocardium susceptible to perforation under sufficient mechanical stress. This supports the observation that hypertrophic myocardium is not absolutely protective against lead penetration.

These findings demonstrate that lead perforation can still occur through the septum and into the LV wall when excessive force is applied or the lead is screwed too deeply. This highlights the need for careful, image-guided lead placement and individualized postprocedural assessment, particularly when the clinical presentation is suggestive of a lead-related complication [4,5,8,10,13,17,18].

The time interval between device implantation and lead perforation ranges from less than 24 hours to as long as nine years [11,18]. Nevertheless, the majority of cases occurred within the first two weeks after implantation, suggesting a relationship with intraoperative factors such as excessive force, lead misplacement, or implantation into weakened myocardial tissue [4,5,9-13,16,17]. In contrast, late-presenting cases are more likely related to chronic myocardial remodeling or degeneration. Clinicians should therefore remain particularly vigilant during the early postoperative period while also recognizing the importance of long-term follow-up in high-risk patients [8].

Clinical presentation varied widely among reported cases. The most common symptoms included chest pain and syncope [4-6,9,11-14,17], which are indicative of myocardial injury. In several cases, symptoms developed within days of implantation, emphasizing key features to monitor during early postoperative surveillance. However, some patients remained asymptomatic, and lead perforation was discovered incidentally during imaging, further underscoring the importance of routine follow-up. Other reported symptoms included bradycardia, dyspnoea, and generalized fatigue [2,6,10,13,15,16,18].

Surgical intervention remains the preferred management strategy in cases of pacemaker or ICD lead-induced septal and LV wall perforation and was employed in the majority of reported cases [4,5,9-18]. Surgical management allows direct visualization and repair of myocardial defects. In the reviewed cases, intraoperative management most commonly involved lead repositioning, either epicardially [10,14,16,18], within the RV septum [5,12,13,17], or at the RV apex [9,15]. In one reported case, transvenous lead extraction was selected due to the patient’s advanced age (92 years) and refusal of open-heart surgery [17], suggesting that non-surgical approaches may be considered in carefully selected, hemodynamically stable patients.

Thus, management should be individualized. Although surgical extraction appears to be the preferred approach, this observation should be interpreted with caution, as published cases are subject to selection bias toward more severe presentations requiring surgical intervention. As summarized in Table 1, surgical management was selected in 11 of the 12 reported cases, underscoring both the potential severity of this complication and the need for tailored decision-making.

## Conclusions

ICD lead perforation extending through the interventricular septum into the LV wall is an exceedingly rare but life-threatening complication. This case involves a young patient, demonstrating that hypertrophic myocardium does not provide absolute protection against myocardial perforation. Careful implantation technique and heightened clinical surveillance after device placement, particularly in the early post-implant period, are therefore essential in patients with hypertrophic cardiomyopathy. Prompt recognition of clinical deterioration, appropriate imaging, and individualized management - most often requiring surgical intervention - are crucial to achieving favorable outcomes. These observations are derived from a single case and should be interpreted with caution, without implying changes to current risk stratification or follow-up practices.
